# Glibenclamide induces apoptosis by activating reactive oxygen species dependent JNK pathway in hepatocellular carcinoma cells

**DOI:** 10.1042/BSR20170685

**Published:** 2017-09-28

**Authors:** Bin Yan, Zhiyong Peng, Xiao Xing, Chunling Du

**Affiliations:** 1 Department of General Surgery, Qingpu Branch of Zhongshan Hospital, Fudan University, Shanghai 201700, China; 2 Department of Respiratory Medicine, Qingpu Branch of Zhongshan Hospital, Fudan University, Shanghai 201700, China

**Keywords:** Apoptosis, Glibenclamide, Hepatocellular carcinoma, JNK pathway, Reactive oxygen species

## Abstract

Glibenclamide (Gli) is a widely employed drug in the treatment of type 2 diabetes and many lines of evidence have described its anti-tumor effects in some neoplasms. The aim of the present study was to investigate the effect of Gli on apoptosis of human hepatocellular carcinoma (HCC) cells and to analyze the underlying pathway involved in this action. Two HCC cell lines, HepG-2 and Huh7 were used as the cell models. We found that Gli treatment significantly inhibited cell viability, induced a significant cell-cycle arrest in G_2_/M-phase and induced apoptosis in both HepG-2 and Huh7 cells. We further verified that apoptosis induction by Gli was accompanied by increase in ROS levels and activation of the JNK pathway. Scavenging of the intracellular ROS with its blocker *N*-acetyl-L-cysteine (NAC) could mitigate the Gli-induced apoptosis and JNK activation in the two HCC cell lines. Furthermore, inhibition of JNK pathway by its inhibitor SP100625 effectively reduced Gli-induced apoptosis in HCC cells. In conclusion, Gli treatment significantly induced cell apoptosis by promoting ROS-dependent JNK pathway activation in HCC cells. Gli may be a potential clinical anti-tumor drug for HCC.

## Introduction

Hepatocellular carcinoma (HCC) is one of the most prevalent malignancies and the third most common cause of cancer-related deaths in the world, accounting for 80–90% of all primary liver cancers [1]. The global incidence of HCC is predicted to keep rising over the next few years, and studies for exploring more effective treatment options are quite urgent.

The combination of unrestrained cell proliferation and impaired apoptosis plays a major role in the progression of HCC tumorigenesis. Apoptosis, also known as programmed cell death, maintains the healthy survival/death balance in metazoan cells. It is executed by apoptotic proteins including released cytochrome *c* (Cyt-*c*) and the activated caspase-3 (cleaved caspase-3) through either extrinsic or intrinsic pathways [2]. Morphological hallmarks of apoptosis include cell shrinkage, chromatin condensation, and DNA fragmentation [3]. Apoptosis is tightly controlled, while defective apoptosis renders cancer cells resistant to treatment and promotes carcinogenesis.

Sulphonylureas, through directly acting on pancreatic β-cells to increase insulin secretion and lower blood glucose concentrations, are widely used in treatment of type 2 diabetes. Mechanistically, these drugs bind to the β-cell sulphonylurea receptor (SUR), a regulatory subunit of ATP-sensitive potassium channels (K_ATP_ channels), and close K_ATP_ channels to reduce the cellular potassium efflux and thus favor membrane depolarization, the induction of Ca^2+^ influx, and insulin secretion [4–6]. Glibenclamide (Gli, also known as glyburide) is a second-generation sulphonylurea, and is widely employed in the treatment of diabetic patients [7,8]. As is known, Gli can block mitochondrial K_ATP_ channels, which play an important role in production of intracellular reactive oxygen species (ROS) [9]. ROS activates both pro- and anti-tumorigenec signaling in cancer cells to control cell growth and apoptosis [10]. This clue raises the possibility that Gli might affect cancer cell survival through ROS generation. Now increasing evidence reveals that Gli exerts anti-tumor effects in many neoplastic cell lines [11,12]. Its genotoxicity in cancer cells or normal cells have been heatingly discussed in recent years, both *in vitro* and *in vivo* [13–15].

Considering the anti-tumoraeffects of Gli on various cancers, we speculated that Gli might also have an affect on HCC cell survival, which has not been addressed before. As an oral anti-diabetic agent, Gli needs to be metabolized by the liver. Therefore, investigating the effects of Gli on HCC cells is of important guiding significance for the medication of clinical diabetic patients in combination with HCC.

## Materials and methods

### Cell culture and reagents

Human HCC cells HepG-2 and Huh7 were from the Cell Resource of Shanghai Institutes for Biological Sciences, Chinese Academy of Sciences (Shanghai, China). Cells were cultured in Dulbecco’s modified Eagle’s medium (DMEM) supplemented with 10% FBS, 2 mM L-glutamine, and PS (100 U/ml and 100 μg/ml) at 37°C in an atmosphere with 5% CO_2_. Gli and *N*-acetyl-L-cysteine (NAC) were purchased from Sigma (St. Louis, MO, U.S.A.). JNK inhibitor SP600125 was purchased from Sellcek (Houston, TX, U.S.A.).

### Cell counting kit-8 assay

Inhibition of cell viability by Gli was measured by using cell counting kit-8 (CCK-8) (Dojindo, Kumamoto, Japan). Cells were seeded in a 96-well-plate at a density of 5000 cells per well containing 100 μl medium. After routine incubation overnight, cells were treated with various concentrations of Gli for another 24 h. Then, the cells were incubated with CCK-8 working solution for 2 h following the manufacturer’s instructions. The resulting absorbance at 450 nm was measured on a microplate reader. Three independent experiments were performed.

### Hoechst 33342 staining

Hoechst 33342 staining was carried out to observe morphological characteristics of apoptotic cells. Briefly, cells were seeded on coverslips in 24-well plates at a density of 5 × 10^4^/ml per well and incubated overnight. Then, the cells were treated with 100 μM Gli for another 24 h. After removal of the medium, cells were washed twice with PBS, fixed with 4% paraformaldehyde for 20 min, permeabilized with 0.1% Triton X-100, and stained with Hoechst 33342 solution (5 μg/ml) for 10 min at room temperature in the dark. Subsequently, the slides were rinsed twice with PBS and photographed with a fluorescent microscope (Olympus Optical Co., Ltd., Tokyo, Japan) for morphological changes in the nucleus.

### Cell cycle analysis

To investigate the cell-cycle attribution influenced by Gli, cells pretreated with Gli (100 µM) for 24 h were trypsinized, washed with PBS, fixed with ice-cold 70% ethanol and incubated overnight at –20°C. Then, the cells were washed with PBS and stained in 0.5 ml of propidium iodide (PI) staining solution (50 μg/ml PI in PBS containing 0.2 mg/ml of DNase free RNase A) for 1 h in darkness at 37°C. Finally, the cells were analyzed by a flow cytometer (Becton Dickinson, U.S.A.).

### Measurement of intracellular ROS generation

Intracellular ROS level was determined by using the Reactive Oxygen Species Assay Kit (Beyotime, Shanghai, China) in which the positive signals of 2′,7′-dichlorfluorescein-diacetate (DCFH-DA) probes reflected ROS generation. Briefly, cells were seeded in a six-well plate at a density of 5 × 10^5^/ml and exposed to Gli (100 μM) for 12 h. According to the protocol provided by the manufacturer, cells were incubated with 10-μM DCFH-DA solution at 37°C for 30 min in darkness, followed by three washes with PBS and flow cytometry analysis of 2′,7′-dichlorfluorescein (DCF) fluorescence.

### Western blotting

Cells were lysed with RIPA buffer (Beyotime, Shanghai, China) containing protease inhibitor cocktail (Sigma) for 30 min on ice, followed by centrifugation at 13000 rpm for 15 min at 4°C. The supernatant was collected and protein concentration was determined by BCA Protein Assay Kit (Beyotime). Equal amounts of whole-cell extracts were heated with SDS sample buffer (Beyotime), loaded on to the 10% SDS/PAGE and electrophoretically transferred to PVDF membrane (Millipore, Billerica, MA, U.S.A.). Membranes were then blocked and incubated overnight with primary antibodies at 4°C individually. Immunoreactivity was detected by using horseradish peroxidase–conjugated anti-mouse or anti-rabbit IgG, and visualized by chemiluminescence. The antibodies were purchased from the following companies: β-actin and the secondary antibodies (goat anti-rabbit and goat anti-mouse; Santa Cruz, Heidelberg, Germany); JNK, p-JNK, uncleaved and cleaved caspase-3/PARP (Cell Signaling, Frankfurt, Germany).

### Annexin V/PI staining

Apoptosis induced by Gli was examined by using the Annexin V-FITC Apoptosis Detection Kit according to the manufacturer’s protocol (KeyGen, Nanjing, China). Briefly, cells were trypsinized, washed with cold PBS, pelleted, and stained using the Annexin V-FITC reaction reagent at room temperature for 20 min in darkness. Then, samples were analyzed by a flow cytometer (Becton Dickinson, U.S.A.).

### Statistics

Data are presented as mean ± S.D. of at least three independent experiments and analyzed using GraphPad Prism 5.0 (Graphpad Software Inc., La Jolla, CA). Student’s *t*test was used for comparison between two groups. *P*<0.05 was considered to be statistically significant.

## Results

### Gli inhibits HCC cell viability and induces G_2_/M arrest in HCC cells

**Figure 1:**
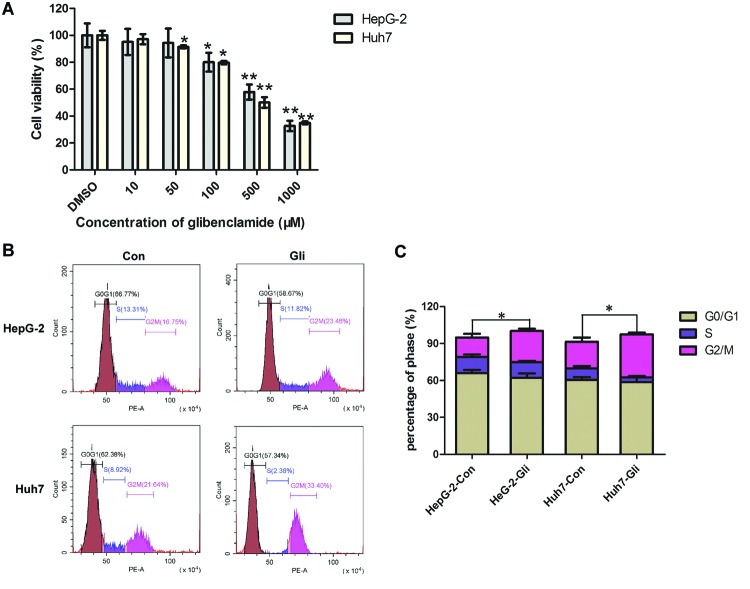
Effects of Gli on HCC cell viability and cell-cycle progression (**A**) HepG-2 and Huh7 cells were treated with different concentrations of Gli (0, 10, 50, 100, 500, 1000 μM dissolved in DMSO) for 24 h. Cell viability was determined by the CCK-8 assay; *P<0.05, **P<0.01. (**B**) HepG-2 and Huh7 cells were treated with Gli (100 μM) for 24 h and cell cycle was analyzed by flow cytometry. Gli caused G_2_/M arrest. (**C**) Quantitation for cell cycle analysis. The percentage of cell population at G_0_/G_1_, S, and G_2_/M phases are represented as mean ± S.D. of three independent experiments; *P<0.05.

In order to verify the effects of Gli on HCC cell survival, two HCC cell line, HepG-2 and Huh7 were treated with different concentrations of Gli for 24 h. Cell viability was assessed by CCK-8 assay. Figure 1A indicates that Gli significantly reduced cell viability in a dose-dependent manner as compared with that noted in the control cells (vehicle treatment with 0.1% DMSO). We observed a significant inhibition on cell viability when Gli was higher than 50 μM for Huh7 cells, and over 100 μM for HepG-2 cells (*P*<0.01–0.05). The analysis of cell-cycle distribution demonstrated that Gli produced a significant arrest in the G_2_/M-phase at 24 h post treatment (*P*<0.05; Figure 1B,C).

### Gli induces cell apoptosis in HCC cells

**Figure 2:**
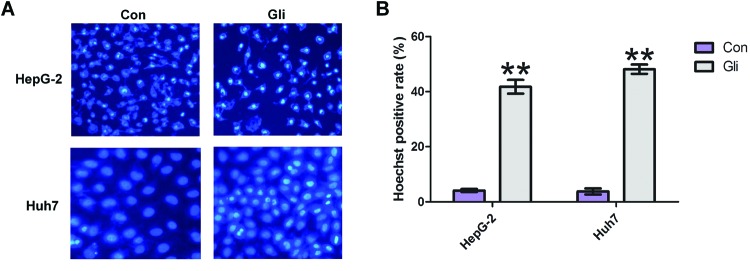
Gli induced cell apoptosis in HCC cells (**A**) Apoptotic nuclear morphological changes were evaluated by Hoechst 33342 staining and observed under a fluorescent microscope. (**B**) The quantitation result of apoptotic cells in (A). The percentage of apoptotic cells was calculated as (highlighted blue staining cell counts)/(total cell counts) × 100; **P<0.01.

To determine if the decrease in cell viability exerted by Gli could be due to apoptosis, we detected cell apoptosis by employing Hoechst 33342 staining. As shown in Figure 2A, Gli-treated cells exhibited different degrees of cell shrinkage, chromatin condensation, and nuclei fragmentation in HepG-2 and Huh7 cells. The quantitation result indicated that Gli induced a significant cell apoptosis (*P*<0.01; Figure 2B).

### Gli increases intracellular ROS level and activates JNK pathway

**Figure 3:**
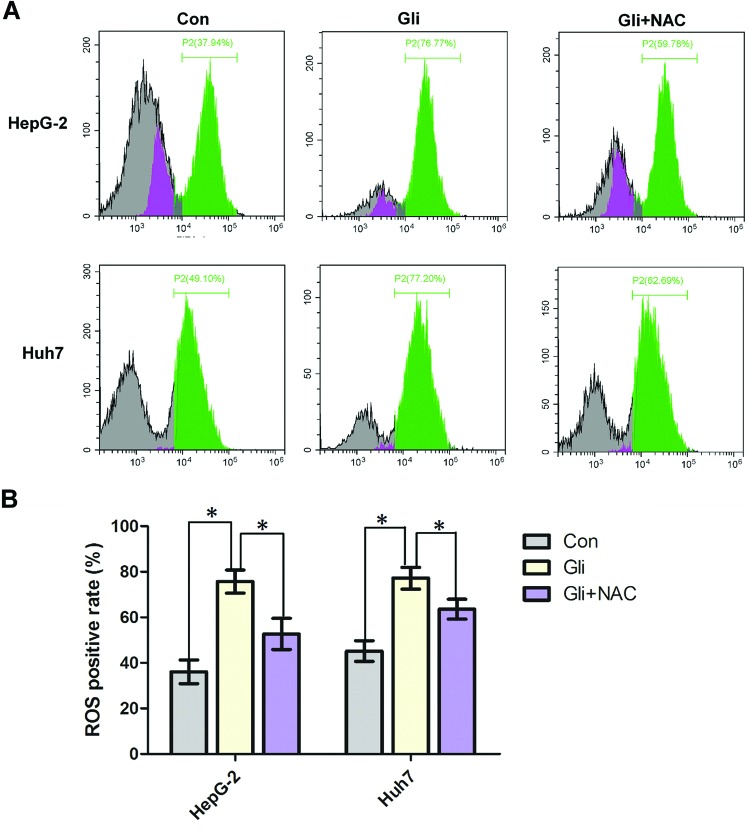
Gli treatment led to intracellular ROS generation (**A**) HepG-2 and Huh7 cells were treated with Gli (100 μM) in presence or absence of NAC (5 mM) for 24 h and intracellular ROS levels were analyzed by DCFH-DA coupled with flow cytometry. Gli caused elevation of ROS levels and NAC pretreatment blocked this action. (**B**) The mean fluorescent intensity of ROS was shown in histograms. Data are presented as means ± S.D. (*n*=3); *P<0.05.

**Figure 4:**
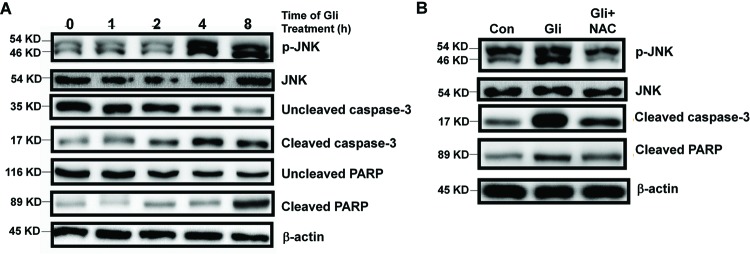
Gli activated JNK pathway in HCC cells (**A**) HepG-2 cells were treated with Gli (100 μM) for indicated hours. Levels of p-JNK, JNK, uncleaved caspase-3, cleaved caspase-3, uncleaved PARP, cleaved PARP, and β-actin were analyzed by Western blotting. (**B**) HepG-2 cells were treated with Gli (100 μM) in presence or absence of NAC (5 mM) for 24 h. Levels of p-JNK, JNK, cleaved caspase-3, cleaved PARP, and β-actin were analyzed by Western blotting.

As mentioned above, through blocking of mitochondrial K_ATP_ channels, Gli plays an important role in production of intracellular ROS, which is an important regulator in various pathways, including apoptosis, and also promotes the sustained JNK activation [16,17]. To investigate whether ROS level was elevated by Gli in HCC cells, DCFH-DA, a fluorescent probe was used to monitor intracellular ROS generation. As shown in Figure 3A,B, cells treated with Gli exhibited a dramatic increase in ROS level compared with control, which was reversed by the ROS scavenger, NAC (*P*<0.05). To further investigate the mechanism of the proapoptotic activity of Gli, we analyzed the effects of Gli on JNK pathway. Figure 4A showed that Gli increased the phosphorylation in JNK in a time-dependent manner. The expression of cleaved caspase-3 or PARP was also up-regulated after 4 or 8 h of Gli exposure, respectively, leaving the decreased amount of uncleaved caspase-3 or PARP, which indicated the occurrence of mitochondrial apoptosis (Figure 4A). We further found that pretreatment with ROS scavenger, NAC, significantly reversed the phosphorylation of JNK, cleaved caspase-3, and PARP in HepG-2 cells (Figure 4B).

### ROS/JNK pathway mediates cell apoptosis induced by Gli

**Figure 5:**
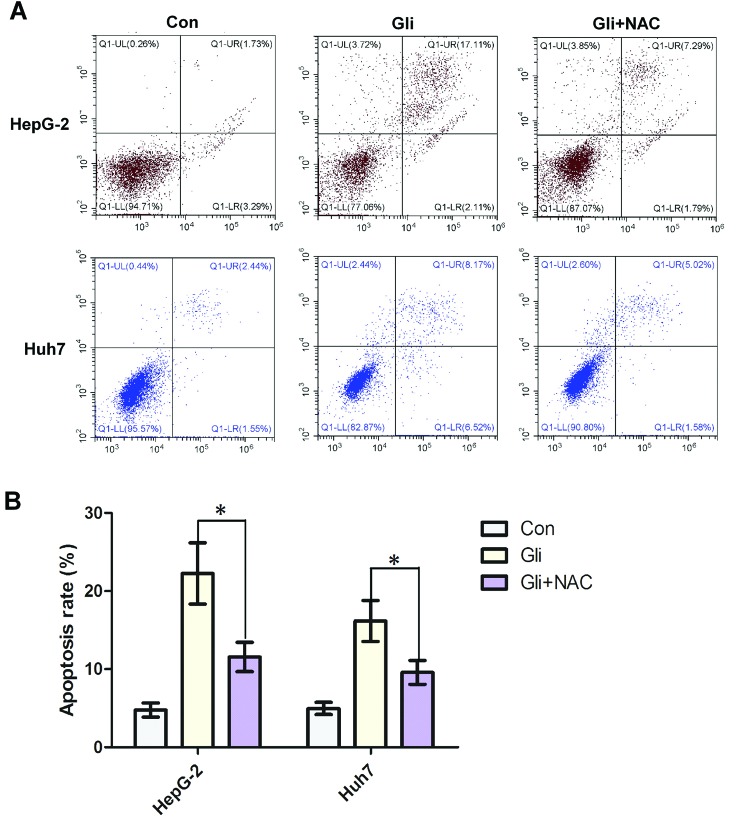
Promotion of cell apoptosis by Gli was ROS dependent (**A**) HepG-2 and Huh7 cells were pretreated with NAC (5 mM) for 2 h and then treated with Gli (100 μM) for 24 h. Cell apoptosis was analyzed by Annexin V/PI staining and flow cytometry. (**B**) Quantitative analysis of apoptosis rate was shown in histograms. Data are presented as means ± S.D. (*n*=3); *P<0.05.

**Figure 6:**
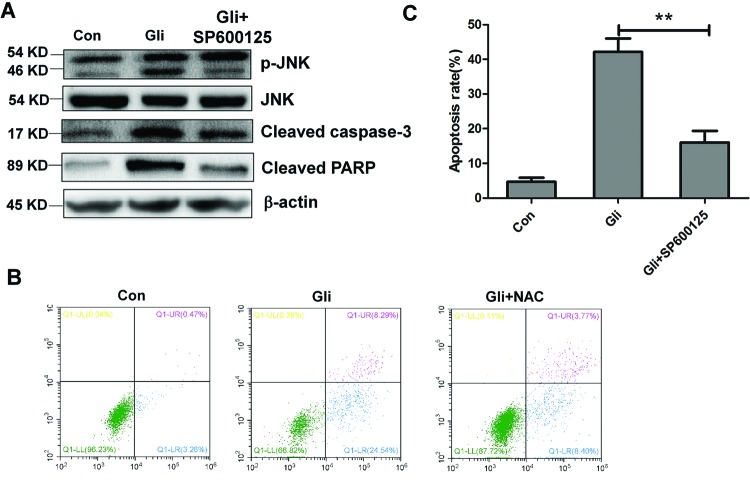
Induction of cell apoptosis by Gli was mediated by JNK activation (**A**) HepG-2 and Huh7 cells were treated with Gli alone or combined with SP100625 (50 nM) for 8 h. Levels of p-JNK, JNK, cleaved caspase-3, cleaved PARP, and β-actin were analyzed by Western blotting. (**B**) Cell apoptosis was analyzed by Annexin V/PI staining and flow cytometry. (**C**) Quantitative analysis of apoptosis in (B) was shown in histograms. Data are presented as means ± S.D. (*n*=3); **P<0.01.

To determine whether Gli-induced apoptosis has a connection with ROS generation, we used NAC, the ROS blocker, for apoptosis investigation. Strikingly, Annexin V/PI staining coupled with flow cytometry analysis revealed that Gli significantly induced HCC cell apoptosis, however, pretreatment of NAC could reverse the apoptosis induced by Gli (*P*<0.05; Figure 5A,B). On the other hand, treatment of JNK inhibitor SP600125 obviously reduced the levels of phosphorylation of JNK, cleaved caspase-3, and PARP (Figure 6A). Consistent with this, Gli combined with SP600125 treatment lowered the apoptosis rate than Gli alone (*P*<0.01; Figure 6B,C). All the above results suggest that HCC cell apoptosis induced by Gli could be mediated by the ROS/JNK pathway.

## Discussion

Gli is a frequently used medicine for treating type 2 diabetes and well recognized for its antiproliferation effects in multiple cancers. For instance, it has been previously demonstrated that Gli inhibits cell growth and by inducing G_1_ arrest in human breast cancer cells [12]. In gastric cancer, Gli induces decline in cell viability, accompanied with cell apoptosis, ROS generation, and JNK activation [11]. There is still no report expounding the effects of Gli on HCC cell survival except that, Gli induces apoptosis through inhibition of cystic fibrosis transmembrane conductance regulator (CFTR) Cl^–^ channels and intracellular Ca^2+^ release in HepG-2 cells [18]. In the present study, we strengthened that Gli could inhibit cell viability, cause G_2_/M arrest, and induce apoptosis in HCC cells. More importantly, we verified that Gli induced apoptosis by activating ROS/JNK pathway.

One of the key findings of the Gli-induced apoptosis in HCC cells is the generation of ROS. ROS is formed as a natural by-product in the metabolism of oxygen and plays critical roles in inducing cell apoptosis [19]. Our results indicated that Gli caused a significant elevation in intracellular ROS level. However, the ROS scavenger, NAC, obviously repressed the ROS, JNK pathway, and apoptosis induced by Gli. This finding illustrated that Gli triggered apoptosis by induction of ROS/JNK pathway.

In HCC cells, we found that Gli treatment increased JNK phosphorylation in a time-dependent manner. Since JNK is generally responsible for the apoptotic response induced by extracellular DNA damaging agents [20], we employed JNK inhibitor SP600125 to observe the Gli’s cytotoxicity. The results showed that SP600125 markedly inhibited Gli-induced apoptosis, accompanied by the reversion of cleaved caspase-3 and PARP. These findings demonstrated that ROS-dependent JNK activation mediated the Gli-induced apoptosis in HCC cells. Thus, the increase in ROS level and JNK activation may naturally sensitize HCC cells to Gli. Application of NAC, the ROS scavenger, could effectively eliminate the ROS/JNK activation and protect HCC cells from Gli-induced cell apoptosis. This is the first work to show the activation of ROS/JNK pathway by Gli treatment in HCC cells, and identifying candidate agents activating ROS/JNK pathway may be a new strategy for HCC chemotherapy.

In conclusion, our study showed that Gli inhibited cell viability by causing G_2_/M arrest and inducing apoptosis in human HCC cells. Additionally, the proapoptotic effect of Gli is mediated by the activation of ROS/JNK pathway. Although *in vivo* data and more detailed mechanisms are still absent from the present study, our data provide further insights into the anti-tumor effects of Gli in HCC cells, and proposes that Gli may be used as a new anticancer drug for HCC.

## Abbreviations

CCK-8: cell counting kit-8
DCFH-DA: 2′,7′-dichlorfluorescein-diacetate
Gli: glibenclamide
HCC: hepatocellular carcinoma
K_ATP_ channel: ATP-sensitive potassium channel
NAC: *N*-acetyl-L-cysteine
PI: propidium iodide
ROS: reactive oxygen species
PS: penicillin–streptomycin
